# Effectiveness of intravenous Dexamethasone versus Propofol for pain relief in the migraine headache: A prospective double blind randomized clinical trial

**DOI:** 10.1186/1471-2377-12-114

**Published:** 2012-09-29

**Authors:** Hassan Soleimanpour, Rouzbeh Rajaei Ghafouri, Aliakbar Taheraghdam, Dawood Aghamohammadi, Sohrab Negargar, Samad EJ Golzari, Mohsen Abbasnezhad

**Affiliations:** 1 Emergency Medicine Department, Tabriz University of Medical Sciences, Daneshgah Street, Tabriz 51664, I.R, IRAN; 2 Neurosciences Research Center, Tabriz University of Medical Sciences, Daneshgah Street, Tabriz 51664, I.R, IRAN; 3 Anesthesiology and Critical Care Department, Tabriz University of Medical Sciences, Daneshgah Street, Tabriz, 51664, I.R, IRAN; 4 Medical Philosophy and History Research Center, Tabriz University of Medical Sciences, Daneshgah Street, Tabriz, 51664, I.R, IRAN; 5 Students’ Research Committee, Tabriz University of Medical Sciences, Daneshgah Street, Tabriz, 51664, I.R, IRAN; 6 Cardiovascular Research Center, Tabriz University of Medical Sciences, Daneshgah Street, Tabriz 51664, I.R, IRAN

**Keywords:** Migraine headache, Propofol, Dexamethasone, Visual Analogue Scale, Emergency Medicine Department

## Abstract

**Background:**

There are many drugs recommended for pain relief in patients with migraine headache.

**Methods:**

In a prospective double blind randomized clinical trial, 90 patients (age ≥ 18) presenting to Emergency medicine Department with Migraine headache were enrolled in two equal groups. We used intravenous propofol (10 mg every 5–10 minutes to a maximum of 80 mg, slowly) and intravenous dexamethasone (0.15 mg/kg to a maximum of 16 mg, slowly), in group I and II, respectively. Pain explained by patients, based on VAS (Visual Analogue Scale) was recorded at the time of entrance to ED, and after injection. Data were analyzed by paired samples *t* test, using SPSS 16. P < 0.05 was considered to be statistically significant.

**Results:**

The mean of reported pain (VAS) was 8 ± 1.52 in propofol group and 8.11 ± 1.31 in dexamethasone group at presenting time (P > 0.05). The VAS in propofol group was obviously decreased to 3.08 ± 1.7, 1.87 ± 1.28 and 1.44 ± 1.63 after 10, 20 and 30 minutes of drug injection, respectively. The VAS in dexamethasone group was 5.13 ± 1.47, 3.73 ± 1.81 and 3.06 ± 2 after 10, 20 and 30 minutes of drug injection, respectively. The mean of reported VAS in propofol group was less than dexamethasone group at the above mentioned times (P < 0.05). The reduction of headache in propofol group, also, was very faster than dexamethasone group (P < 0.05). There were no adverse side effects due to administration of both drugs.

**Conclusions:**

Intravenous propofol is an efficacious and safe treatment for patients presenting with Migraine headache to the emergency department.

**Trial registration:**

Clinical Trials IRCT201008122496N4

## Background

Headache is one of the common complaints more frequent than common cold. It allocates almost 3 million visits to the Emergency Department (ED) in the United States. Most of patients presenting with headache have benign headaches that need symptomatic treatments [1]. Migraine typically starts in the 2nd decade of life, reaches its peak in the middle age, and its prevalence among women (18%) is more than among men (6%) [2,3]. The lifetime prevalence of migraine is at least 18% [4]. In a study carried out on 405 patients with primary chronic headache, it was revealed that 95% of the patients complained of tension type headache and only 4% of them suffered from chronic migraine headache [5].

The International Headache Society (IHS) has defined the migraine headache criteria related to the migraine headache as following:

A. At least five attacks fulfilling criteria in B, C, D, and E.

B. Attack lasts 4 to 72 hours with or without treatment.

C. Headache has at least two of the following characteristics:

1. Unilateral location

2. Pulsating quality

3. Moderate to severe intensity

4. Aggravated by walking up stairs or similar routine physical activity

D. During headache, at least one of the following:

1. Nausea or vomiting (or both)

2. Photophobia and phonophobia

E. History, physical and neurologic examination and, if appropriate, diagnostic tests to exclude related organic disease [1]

This society (IHS) has introduced acetaminophen and NSAIDs for mild to moderate cases of migraine and in more severe or refractory and status cases selective receptor agonists 5HT (triptans category) and steroids have been introduced [6,7]. In some studies, there has been a 49% relapse after migraine treatment leading to exacerbation of anxiety in patients in EDs [8,9].

A new drug that few studies have been carried out regarding its role in relieving migraine headaches is propofol (2 and 6 di- isopropyl phenol). Its pharmacological mechanisms are related to its agonist characteristics on gamma-amino butyric acid (GABA) receptors [10]. The drug also inhibits the afferent sympathetic activity and reduces reflex sensitivity of the cardiac baroreceptor reflex [11]. Propofol can also cause vasodilatation by stimulating production of nitric oxide (NO) [12].

In this study we tried to investigate the role and effect of intravenous administration of propofol in migraine headache relief compared with one of the other current treatments (intravenous dexamethasone) in patients who presented to ED of Imam Reza teaching hospital in Tabriz, Iran.

## Methods

**Figure 1:**
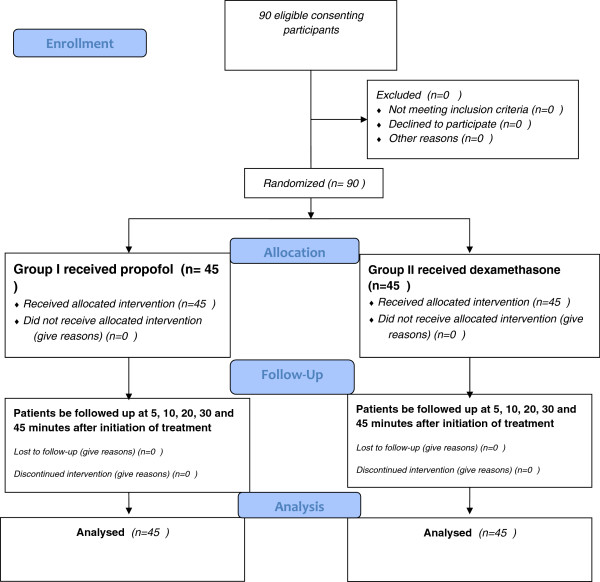
CONSORT 2010 Flow Diagram of trial design.

A clinical trial study was performed in the ED of Imam Reza teaching hospital, Iran [13]. Based on existing studies and articles, and in particular with regards to the results obtained from the study of Krusz and colleagues reporting a 95% efficacy of propofol in the treatment of migraine headache [10], 38 samples of each group were determined considering α = 0.05, p =%95 and d = 0.06 that 45 samples for each group were examined and determined to increase validity of the study. Therefore, the study population was considered 90 people (age ≥ 18). First patients who were complaining of headaches were detected, and then history was taken and complete examination (general and neurologic examination) of the patients was performed. So that based on IHS criteria migraine diagnosis has been established [1]. The full trial design is summarized in Figure 1.

Patients with the following conditions were excluded:

– History of receiving opioid compounds or other triptans such as vasoconstrictors like dihydroergotamine 24 hours before referring to the emergency.

– Patients treated with systemic corticosteroids

– Allergy to propofol and dexamethasone, or eggs and soy.

– Patients with a history of Diabetic Mellitus, active peptic ulcers, Myocardial Infarction within the last week and familial hypokalemic periodic paralysis (for dexamethasone).

This study was approved by Ethics Committee of “Tabriz University of Medical Sciences”. However, all aspects of the present study plan were explained to patients and then we obtained their written consent.

Patients were randomly divided into two equal subgroups I and II each with 45 people by picking a ballot. Group I received propofol and group II received dexamethasone. Variables examined in the study were: headache intensity based on VAS value criteria, before treatment and at 5, 10, 20, 30 and 45 minutes after treatment, age and sex.

Using a graded page that was numbered from 0 to 10; the patients were asked to mark the page based on the pain they tolerated in which 0 is the sign of analgesia and 10 is the sign of the most severe pain. Patient's vital signs and other signs such as nausea, vomiting, photophobia, phonophobia were recorded. After establishing intravenous line, patients were connected to cardiac monitors, automated blood pressure monitors, pulse-oximeters and capnography monitors. Another specialist who was blinded to all procedures of the study filled out the questionnaires and performed the injections. All injections were performed on the left hand of the patients. To blind the injection performer to the type of injection, a curtain separating the hands and upper body of the patient from the lower part was utilized to prevent the performer from distinguishing the injection site (the curtain was attached to two tall vertical stands lying on both sides of the patient).

Patients were blinded to the injected medications as well. In group I propofol (LIPURO-B.BRAUN) 1% was injected intravenously every 5 to 10 minutes with bolus dose of 10 mg (maximum dose of 80 mg) slowly (at the rate of 1 mL over 10 seconds), until pain was maximally relieved (VAS ≤ 2) [1].

With the same method in Krusz’s study, the treatment response appeared at 50 mg minimally and at 110 mg in maximally as well [9]. To avoid pain at injection site 1 mL of Lidocaine 2% per 10 mL of propofol was added. It has already been identified that giving such a dose of lidocaine (1 mL of lidocaine 2%) even as intravenous bolus has no effect on reducing headache [9]. In group II intravenous dexamethasone (Decardol) 4 mg/ml with dose of 0.15 mg/kg (maximum dose of 16 mg) was injected, slowly (at the rate of 1 mL over 10 seconds).

Based on VAS scaling, level of headache, 5, 10, 20, 30 and 45 minutes after initiation of treatment was recorded in patients. When no improvement in pain of each of two groups was observed, common medications such as opioids and NSAIDs were used.

Data obtained from the study were statistically analyzed by descriptive statistics (mean ± SD), analysis of variance and repeated measures (Repeated Measurement) and statistical software SPSS 16. In this study, P values less than 0.05 was considered significant.

## Results

The mean age of patients treated with dexamethasone was 36.27 ± 13.38 and in group treated with intravenous propofol was 35.65 ± 12.55 years. Independent *T*-test results showed no significant difference in terms of average age in the two study groups (P = 0.832).

In dexamethasone-treated group there were 28 females (62.22%) and 17 males (37.77%) and in the propofol-treated group there were 30 females (66.6%) and 15 males (33.3%). Chi-square test results showed no significant difference in terms of gender distribution in the two study groups (p = 0.577).

Considering associated symptoms, the findings of this study are as follows:

1. Nausea: most common symptom associated with both groups of patients with headache was nausea. The prevalence of nausea in the group treated with dexamethasone and propofol-treated group was 95.55% and 93.33% respectively.

2. Vomiting: Seventeen patients in the group treated with dexamethasone (37.77%) and 18 cases of patients treated with propofol group (40%) had this accompanying symptom.

3.Photophobia: Fourteen cases of patients in the group treated with dexamethasone (31.11%) and 17 cases of patients treated with propofol group (37.77%) complained of photophobia.

4. Phonophobia: Nine patients in the group treated with dexamethasone (20%) and eight cases of patients treated with propofol group (17.77%) complained of this symptom.

Chi-square test results showed no significant difference (P > 0.05) on the prevalence of the above-mentioned symptoms in groups under study.

Average pain in the group treated with dexamethasone prior to the desired intervention was 8.11 ± 1.31 and it was 8 ± 1.52 in groups treated with propofol. The independent *T*-test results showed no significant difference in terms of pain severity in both groups on arrival and before any therapeutic intervention (P = 0.712).

Pain level was compared in patients using the VAS method in times of entering the study and at 5, 10, 20, 30 and 45 minutes after intervention in both groups who were treated with dexamethasone and propofol.

Table 1 compares the level of pain reduction at different times after treatment interventions in the groups treated with dexamethasone and propofol. As it can be seen, level of pain reduction has significantly decreased more at all times in patients treated with propofol (P < 0.001).

**Table 1:** **Comparing the rate oftreatment response between twogroups**

	**At presenting time**	**Minute 5**	**Minute 10**	**Minute 20**	**Minute 30**	**Minute 45**
VAS in Group I (Propofol)	8 ± 1.52	5.46 ± 1.56	3.08 ± 1.7	1.87 ± 1.28	1.44 ± 1.63	1.16 ± 1.55
VAS in Group II (Dexamethasone)	8.11 ± 1.31	6.57 ± 1.57	5.13 ± 1.47	3.73 ± 1.81	3.06 ± 2	2.87 ± 1.81
P Value	0.712	0.001	<0.001	<0.001	<0.001	<0.001

Although comparing intra-group pain level mean within different times revealed that the pain level decreased from 8 to 1.8 in minute 20 in groups treated with propofol, the group treated with dexamethasone never improved to this extent.

In addition to evaluated and compared pain level at different times in the two study groups, pain reduction was assigned in every individual group over time and the level of their significance was evaluated. Findings of this comparison are given in Tables 2 and 3. Pain reduction was higher than other times in the first 5 minutes and the rate of pain reduction decreased over time.

**Table 2:** **The average of painreduction at different timesin patients received Propofol**

**The pain score in…**	**The average of painreduction in group I**	**P Value**
Minute 5 comparing with presenting time	2.53 ± 1.34	<0.001
Minute 10 comparing with minute 5	2.37 ± 1.41	<0.001
Minute 20 comparing with minute 10	1.41 ± 1.48	<0.001
Minute 30 comparing with minute 20	0.827 ± 1.25	0.001
Minute 45 comparing with minute 30	0.375 ± 0.646	0.009

**Table 3:** **The average of painreduction at different timesin patients received Dexamethasone**

**The pain score in…**	**The average of painreduction in group II**	**P Value**
Minute 5 comparing with presenting time	1.53 ± 1.07	<0.001
Minute 10 comparing with minute 5	1.44 ± 1.25	<0.001
Minute 20 comparing with minute 10	1.4 ± 1.37	<0.001
Minute 30 comparing with minute 20	0.177 ± 3.02	0.695
Minute 45 comparing with minute 30	0.45 ± 0.782	0.001

Based on the findings contained in Table 2, as can be seen pain reduction in patients treated with propofol in the first 5 minutes is also more than other times. In this group, pain reduction in 2nd five minutes is high as well. Like the group treated with dexamethasone, pain reduction decreased over time. The difference is that pain reduction was in a higher rate in this group.

Comparing the rate of treatment response in both groups, as can be seen in Table 1, in groups treated with propofol in 10 minutes, revealed that pain level mean decreased from 8 to 3.08. Whereas in the dexamethasone treated group, 30 minutes were needed for the pain level mean to reach the above mentioned number. Although no significant difference was observed in terms of pain level mean after 20 minutes, mean pain was always lower in propofol treated group.

In this study, the parameter of good response to treatment is considered as VAS ≤ 2 [1]. Accordingly, in none of the defined periods was there significant difference in terms of response to treatment between males and females in both groups treated with propofol and dexamethasone (P > 0.05).

Over time, in the tenth minute in patients treated with propofol, the response to treatment was considerably high (40% comparing with 2% in patients treated with dexamethasone). The highest rate of response in the group treated with dexamethasone was in minute 30 (51%) and in propofol treated group at minute 20 (66%).

Mean blood pressure, heart rate and O_2_ Saturation of patients were compared in defined times in both groups and there was no significant difference. Then the patients were followed up in the hospital until they were discharged as following: In 20 cases of patients treated with propofol (44.4%) mild sedation was observed as a complication. Also, slurred speech was reported in one patient and in two cases mild decline in arterial oxygen saturation (O_2_ Saturation = 89%) was reported which was quickly resolved with nasal oxygen administration.

## Discussion

Since migraine is a prevalent disease, several studies have been carried out in the world on how to treat severe migraine headaches in patients referred to emergency departments. A study concluded that the treatment of refractory migraine requires aggressive intravenous treatment [14]. In a recent study on the health care cost in the patients with migraine headache, it was suggested that 45% of the patients with migraine headache do not receive appropriate treatment. Total health care cost tends to be higher in these patients due to their frequent out-patient and emergency referrals compared to the ones receiving medications regularly, even expensive anti-migraine agents [15].

Although many researches have been carried out on the effectiveness of propofol pain relief after surgery, few studies have been conducted in order to evaluate its efficacy in relieving pain in the emergency department.

As mentioned in results, the study groups were identical and based on VAS scoring criteria had no significant difference regarding age, sex and pain level. Although both dexamethasone and propofol are effective in relieving headache over time, propofol is more effective than dexamethasone and at different times, pain reduction is significantly higher in propofol treated patients than patients treated with dexamethasone.

Many studies have discussed the efficacy of dexamethasone. In most of the studies it is noted that dexamethasone, especially in combination with other drugs, has considerable effect on migraine. The findings of this study, like most studies, confirm this. But some studies have rejected the efficacy of dexamethasone [16] and others have reported it to be beneficial in the treatment of migraine by adding it to other common drugs [17-19]. Friedman and his colleagues compared dexamethasone with placebo. They showed that there was no significant difference between the two groups at first hour regarding pain reduction (P = 0.03) and they did not recommend routine use of intravenous dexamethasone [20]. Other studies have been carried out on the role of dexamethasone in the relapse rate of migraine headache. Some of these studies have shown that dexamethasone reduces migraine relapse in patients [21]. A study also showed that dexamethasone reduced relapse by 50% and compared with NSAIDs and triptans, in addition to controlling it also reduces relapse of headache intensity [22]. Other studies have emphasized on the effective role of dexamethasone in reducing recurrent migraines [23,24]. However, some have questioned it and despite the findings of this study considered it an ineffective drug [25].

Unlike dexamethasone, few studies have been conducted on the role of propofol, the most common medication used in anesthesia induction in operating room, in the treatment of migraine headache [26,27].

In 2002 two cases of migraine were reported to be treated by intravenous propofol in which the headache scoring of the first and the second patients dropped from 100/100 to 10/100 and from 92/100 to 40/100 respectively [28].

In another study of ours carried out on 8 patients with refractory migraine, propofol administration significantly reduced migraine headache in the patients [29]. In the current study, the average rate of pain score according to VAS reduced from 8/10 to 1/10. On the other hand in the present study, rate of pain reduction has been considered by researchers. Comparing the treatment response rate in both groups, the average pain level in propofol group declined from 8 to 3.08 in the tenth minute. While in the dexamethasone group, after 30 minutes, the average pain level reached the above-mentioned number. Therefore, the treatment response rate was considerably higher in the propofol treated group. Other reports have been published on the use of intravenous propofol at sub-hypnotic dose for refractory migraine [30].

As mentioned before, rate of response to treatment in this study was defined and evaluated as VAS ≤ 2. The highest rates of response to treatment were recorded in the 10th and 20th minutes by both drugs. The difference was that the rate of response to treatment in these times was considerably higher in the propofol treated group.

Like our findings, results of another study that is the largest study on the role of propofol in the treatment of headache showed that 82% of 77 patients with severe headache (between 7–10) who were scored with VAS, were completely pain-relieved and in the rest of them pain declined by 50 to 90 percent [10]. Another study has recommended the administration of propofol for chronic daily headache [31].

In most of studies, the main reason of propofol’s remarkable effect is reported as the high tendency of propofol to GABA receptors that are in low functional status in this disease so that propofol overcomes them through stimulating in this physiological process. Researchers have asserted that using other drugs with this property (excitatory GABA receptors) as potential drugs to treat migraine and other headaches, requires further investigation [11].

It seems that propofol plays its therapeutic role affecting chlorine channels in β1 subunits of GABA receptors [29,32]. Medication Overuse Headache (MOH) is a term frequently used in association with chronic migraine; it however can be used in cases with over usage of all medications used for headache treatment as well [33]. Propofol not only, similar to all other medications, might cause MOH but also could be considered an effective treatment of MOH. Therefore, further studies focusing on the comparison of the probable therapeutic effects of propofol and other conventional medications including Topiramate and onabotulinumtoxinA are required to be conducted.

### Limitations

Similar to most of ED trials, our sampling was convenience; therefore we might have had an unrecognized selection bias. In addition, we did not select a standard abortive treatment as different drug combinations are often required and not all patients do respond to a standard regimen.

Although all patients were discharged after pain was relieved, we did not follow up the patients in days after being discharged from ED. However, one of the merits of IV Propofol and Dexamethasone is that their effect may be prolonged, even after a single bolus administration. Patients may get hours, days or weeks of relief. It seems necessary to evaluate the patients in the following days, as the question of whether the pain relief period is extended after discharge still remains. If so, then for how many days would this period last?

Furthermore we did not assess and compare the rate of relapse in both groups.

Our study shows that dexamethasone and specially propofol are useful in the acute migraine headache but does not tell us whether they decrease recurrent headache. And also we have no data on several abortive agents; we cannot draw any conclusion on which is the most effective regarding the relief of acute headache.

Another limitation of our study was to administer titrated doses of propofol whereas dexamethasone was administered in bolus form. This was due to the fact that there is no medication effective in migraine treatment used titrated intravenously so that it could be compared as a conventional treatment with propofol. Furthermore, common intravenous treatments for migraine including NSAIDs and sumatriptan are not available in our country; therefore, propofol was selected to be compared with dexamethasone.

Although our study suggested propofol as a new and effective treatment which may improve the quality of life and have productivity benefits to this therapy, we did not perform a formal economic analysis demonstrating these benefits.

All above-mentioned items are areas requiring further investigations in the future.

## Conclusion

According to findings of this study, the speed and rate of response to migraine headache treatment is considerably higher using propofol than dexamethasone and pain relief in patients treated with propofol improves more quickly based on the VAS. This drug has also no considerable side effect and can therefore be administered as an effective medication with low side effect and good availability to treat migraine headaches.

## Abbreviations

VAS: Visual Analogue Pain Scale; NSAIDs: Nonsteroidal anti-inflammatory drugs; ED: Emergency medicine Department; MOH: Medication Overuse Headache.

## Competing interests

The authors declare that they have no competing interests.

## Authors' contributions

HS, AAT and RRG collected clinical data, reviewed the literature on the topic and drafted the manuscript. DA, SN, MA and SEJG analyzed and interpreted the patient data. All of the authors were involved in patient management or the writing of the manuscript. All authors read and approved the final manuscript.

## Authors information

HS is Associate professor of Anesthesiology and Critical Care, Fellowship in Trauma Critical Care and CPR at the Department of Emergency Medicine, Tabriz University of Medical Sciences, Tabriz, Iran. He is also editorial board member of Emergency medicine journal (EGM) and Pakistan Journal of Biological Sciences (PJBS). RRG is Assistant professor of Emergency Medicine at the Department of Emergency Medicine, Tabriz University of Medical Sciences, Tabriz, Iran. AAT is Associate professor of Neurology at the Department of Neurology, Tabriz University of Medical Sciences, Tabriz, Iran. DA and SN are Assistant professor of Anesthesiology and Critical Care and Associate professor of Anesthesiology and Critical Care at the Department of Anesthesiology and Critical Care, Tabriz University of Medical Sciences, Tabriz, Iran, respectively. SEJG is resident of Anesthesiology and Critical Care at the Department of Anesthesiology and Critical Care, Tabriz University of Medical Sciences, Tabriz, Iran. MA is internist (subspecialty in cardiology) and Assistant professor of Cardiology at the Department of Cardiology, Tabriz University of Medical Sciences, Tabriz, Iran.

Funding International Clinical Trials Registry Platform (ICTRP).

## Pre-publication history

The pre-publication history for this paper can be accessed here:

http://www.biomedcentral.com/1471-2377/12/114/prepub
